# Association between muscular tissue desaturation and acute kidney injury in older patients undergoing major abdominal surgery: a prospective cohort study

**DOI:** 10.1007/s00540-024-03332-6

**Published:** 2024-04-06

**Authors:** Lingzi Yin, Chunsheng Wang, Wanli Zhao, Xiaoxia Yang, Yuhao Guo, Dongliang Mu, Xinli Ni

**Affiliations:** 1 Department of Anesthesiology and Perioperative Medicine, General Hospital of Ningxia Medical University, No.804 Shengli Street, Yinchuan, 750001 Ningxia China; 2 Department of Anesthesiology, Peking University First Hospital, Beijing, China

**Keywords:** Muscular tissue desaturation, Acute kidney injury, Abdominal surgery, Older patients

## Abstract

**Purpose:**

Present study was designed to investigate the association between muscular tissue desaturation and acute kidney injury (AKI) in older patients undergoing major abdominal surgery.

**Method:**

A total of 253 patients (≥ 65 years old) who underwent abdominal surgery with expected duration ≥ 2 h were enrolled. Muscular tissue oxygen saturation (SmtO_2_) was monitored at quadriceps and bilateral flanks during surgery. Muscular desaturation was defined as SmtO_2_ < 90% baseline lasting for > 60 s. The primary outcome was the incidence of AKI within postoperative 7 days. The association between muscular desaturation and AKI was analyzed by multivariable logistic regression model. The secondary outcomes indicated the other complications within postoperative 30 days.

**Results:**

Among 236 patients, 44 (18.6%) of them developed AKI. The incidence of muscular desaturation at quadriceps was 28.8% (68/236). Patients with muscular desaturation had higher incidence of AKI than those without desaturation (27.9% [19/68], vs. 14.9% [25/168], *P* = 0.020). After adjustment of confounders, multivariable analysis showed that muscular desaturation at quadriceps was significantly associated with an increased risk of AKI (OR = 2.84, 95% CI 1.21–6.67, *P* = 0.016). Muscular desaturations at left and right flank were also associated with an increased risk of AKI (OR = 6.38, 95% CI 1.78–22.89, *P* = 0.004; OR = 8.90, 95% CI 1.42–45.63; *P* = 0.019, respectively).

Furthermore, patients with muscular desaturation may have a higher risk of pulmonary complications, sepsis and stroke at 30-day follow-up.

**Conclusion:**

Muscular desaturation was associated with postoperative AKI in older patients undergoing major abdominal surgery which may serve as a predictor of AKI.

**Supplementary Information:**

The online version contains supplementary material available at 10.1007/s00540-024-03332-6.

## Introduction

Acute kidney injury (AKI), characterised by a sudden elevation of serum creatinine levels, with or without the presence of oliguria, is a major complication in patients undergoing abdominal surgery with an incidence of 6.3% to 13.4% [1, 2]. Older patients are prone to suffer AKI because of age-dependent deterioration in renal structure and function [3]. AKI is associated with poor short-term and long-term outcomes including prolonged hospitalisation, increased risk of readmission, and mortality [4–6].

One of the primary contributors to AKI is renal hypoxia, particularly in the medulla of the kidney [7, 8]. Several indicators have been proposed to reflect renal oxygenation such as oxygen delivery index and urinary oxygen tension [9, 10]. Nevertheless, direct monitoring of renal oxygenation remains a formidable challenge. Near-infrared spectroscopy (NIRS) offers a non-invasive and continuous approach to monitor oxygenation balance in regional tissues [11]. Recent studies focusing on infants undergoing cardiac surgery have illuminated the potential of intraoperative renal desaturation monitored by NIRS as a predictor of increased AKI risk [12, 13]. However, when it comes to adults, the feasibility of directly monitoring renal saturation is hampered by the presence of thicker subcutaneous tissue layers, which extend beyond the detection capability of NIRS probes**.**

Muscular tissue oxygen saturation (SmtO_2_) serves as a valuable indicator reflecting the balance between oxygen consumption and supply in the skeletal muscle [14]. It can be intraoperatively measured at certain sites such as quadriceps and flanks. Several studies have shown that a decline of SmtO_2_ not only represents insufficient tissue perfusion, but also is related to several adverse complications and mortality [15].

A previous study demonstrated that monitoring SmtO_2_ of thenar muscle below 75% is an early indicator of impaired lactate clearance in the first hour after surgery [16]. Another study found that SmtO_2_ was associated with well-known perioperative risk factors for morbidity and mortality [17]. In adults undergoing major spine surgery, SmtO_2_ had shown stronger associations with both the duration of hospitalization and postoperative composite complications [18]. A reduction of SmtO_2_ was identified as a risk factor of postoperative nausea and vomiting in patients undergoing robotic hysterectomy [19]. However, whether muscular tissue desaturation is associated with an increased incidence of AKI in older patients after abdominal surgery remains uncertain. In this study we aimed to investigate the association between muscular tissue desaturation and AKI in older patients after major abdominal surgery.

## Methods

### Study design

This prospective cohort study was approved by the Clinical Research Review Board of Ningxia Medical University (No. KYLL-2021-465) on 28 June 2021 and registered at Clinical Trial Registry (No. NCT04954066) on 8 July 2021. This study was conducted at the General Hospital of Ningxia Medical University from September 2021 to August 2022. Written informed consent was obtained from all participating patients. The manuscript adheres to the Strengthening the Reporting of Observational Studies in Epidemiology (STROBE) reporting guidelines.

### Participants

Patients ≥ 65 years old who underwent elective major abdominal surgery (expected duration ≥ 2 h) were enrolled. The exclusion criteria were as the following: (i) refusal to participate; (ii) body mass index (BMI) ≥ 30 kg/m^2^; (iii) pre-operative glomerular filtration rate less than 30 ml/min/1.73m^2^; (iv) a history of nephrectomy or partial nephrectomy; (v) administration of contrast agent within 24 h before surgery; (vi) skin abnormalities precluding the placement of oximetry probes; and (vii) impaired hearing or vision impeding communication.

### Perioperative anesthetic management

All patients received the standard monitoring, including electrocardiogram, pulse oximetry, invasive radial artery pressure, bispectral index (BIS), nasopharyngeal temperature, and end-tidal carbon dioxide (EtCO_2_). Advanced hemodynamic parameters (i.e. cardiac index and stroke volume variation) were monitored in terms of the patient’s condition.

Total intravenous anesthesia was administered to all patients, with the option of epidural or transverse abdominal fascia block determined by the attending anesthesiologists. Anesthesia was inducted with sufentanil and etomidate, followed by maintenance with propofol and remifentanil to maintain BIS values between 40 and 60. Mechanical ventilation was maintained with an inspired fraction of oxygen at 50%, a tidal volume of 6–8 ml/kg, and peripheral oxygen saturation (SpO_2_) was maintained above 92%, with end-tidal carbon dioxide (EtCO_2_) between 35 and 45 mmHg. Nasopharyngeal temperature was maintained at 36–37 °C. Intraoperative mean arterial pressure (MAP) was maintained ≥ 60 mmHg. Autologous or allogeneic red blood cell was transfused when the haemoglobin level < 7 g/dL.

### Tissue oxygenation monitoring

SmtO_2_ was non-invasively monitored using a tissue oximeter based on NIRS (EGOS-600A, ENGIN, Suzhou, China). One probe was placed over the lateral distal end of the quadriceps muscle, while two additional probes were parallelly placed to the bilateral paraspinal muscle (2 cm beside the spine at the T12–L1 level) to monitor oxygen saturation at the right and left flanks, respectively.

Baseline value was measured when patient was resting and breathing room air before anesthesia induction. SmtO_2_ was continuously monitored throughout the surgery, with data captured at 2-s intervals. To ensure uninterrupted monitoring, the tissue oximeter screen was covered, and designated research personnel conducted periodic checks every 10 min to confirm its proper functionality.

### Postoperative AKI

The primary outcome was the incidence of AKI within postoperative 7 days. Serum creatine (SCr) was measured at one day before surgery and within postoperative first 7 days. AKI was diagnosed by acute change of serum creatine level according to the criteria of Kidney Disease: Improving Global Outcomes (KDIGO) [20]. Severity of AKI was divided into stage 1 (an increase of 0.3 mg/dl or greater in the SCr level within 48 h), stage 2 (a 1.5-fold or higher increase from the baseline SCr level within 7 days), and stage 3 (requirement of dialysis).

### Other complications

All patients were followed up within 30-days postoperatively to observe other complications included pulmonary complications, cardiac complications, stroke, sepsis, incision infection, pulmonary embolism, deep vein thrombosis, hepatic dysfunction, duration of hospitalization, 30d-rehospitalization, 30-day mortality and patient’s recovery quality.

### Sample size

In patients undergoing cardiac surgery, intraoperative SmtO_2_ less than 80% baseline was associated with 2.9-fold risk of AKI (95% CI 1.2 to 7.2) [21]. We assumed a similar association between muscular desaturation and AKI in non-cardiac patients with an estimated incidence of muscular tissue desaturation of approximately 25% in patients undergoing major non-cardiac surgery [19]. Considering statistical significance at 0.05 and power at 0.8, 230 patients were needed to detect difference. To account for a potential dropout rate of 10%, 253 patients were planned for enrolment.

### Statistical analysis

Normality was tested by Q–Q plot. Continuous variables with normal distribution were reported as mean ± standard deviation and compared by independent t test; otherwise, they were presented as median (interquartile range) and compared by Mann–Whitney U test. Categorical variables were reported as number (percentage) and compared by chi-square or Fisher exact test.

Minimum SmtO_2_ was defined as the absolute lowest value lasting for more than 60 s during surgery. The relative change of SmtO_2_ was calculated as the differences between baseline value and minimum SmtO_2_. Initially, unadjusted restricted cubic spline (RCS) models were employed to visualize the association between minimum and relative change of SmtO_2_ at different tissue beds with AKI. The threshold for desaturation was selected based on the abrupt change in SmtO_2_ corresponding to an odds ratio of 1 in the RCS curve (i.e., < 90% of baseline). This threshold was subsequently tested using a multivariable regression model.

According to the threshold, patients were divided into desaturation group and normal group. For primary outcome, the incidence of AKI was presented as number (percentage). Differences between the groups was compared by Chi-square test. The confounders in multivariable logistic regression were selected according to clinical risk factors and univariate analysis. The association between muscular desaturation and AKI was analyzed by multivariable logistic regression. We additionally analyzed the associations between the AUCs (Area under curve) at different relative thresholds and AKI by using the same multivariable logistic regression. AUCs (min × %) were calculated as the area accumulated throughout the period when the actual measurement exceeded the given threshold.

The incidence of other postoperative complications and 30-mortality between the groups were compared by chi-square, while the length of postoperative in-hospital stay was tested by Mann–Whitney U test between groups. Statistical analysis was performed using SPSS 26 (IBM, Inc. Chicago, IL, USA), R (v4.3.1, R Foundation for Statistical Computing, Vienna, Austria) and GraphPad Prism (version 9.0, GraphPad Software Inc, San Diego, CA, USA). *P* < 0.05 was considered as statistical significance.

## Results

### Patient characteristics and perioperative data

**Fig. 1:**
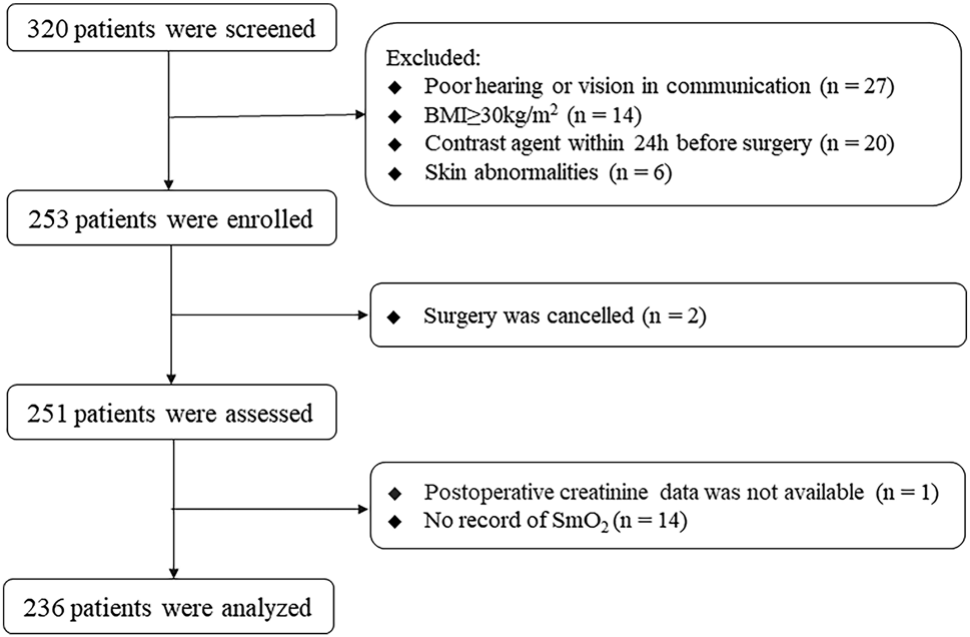
Flowchart of the study. AKI, Acute kidney injury; SmO_2_, Muscular tissue oxygen saturation

During the period from September 2021 to August 2022, a total of 320 patients were initially screened, with 253 ultimately enrolled in the study. Seventeen patients were excluded due to reasons including surgery cancellation (n = 2), absence of postoperative creatinine data (n = 1), or lacking SmtO_2_ records (n = 14), Fig. 1.

**Fig. 2:**
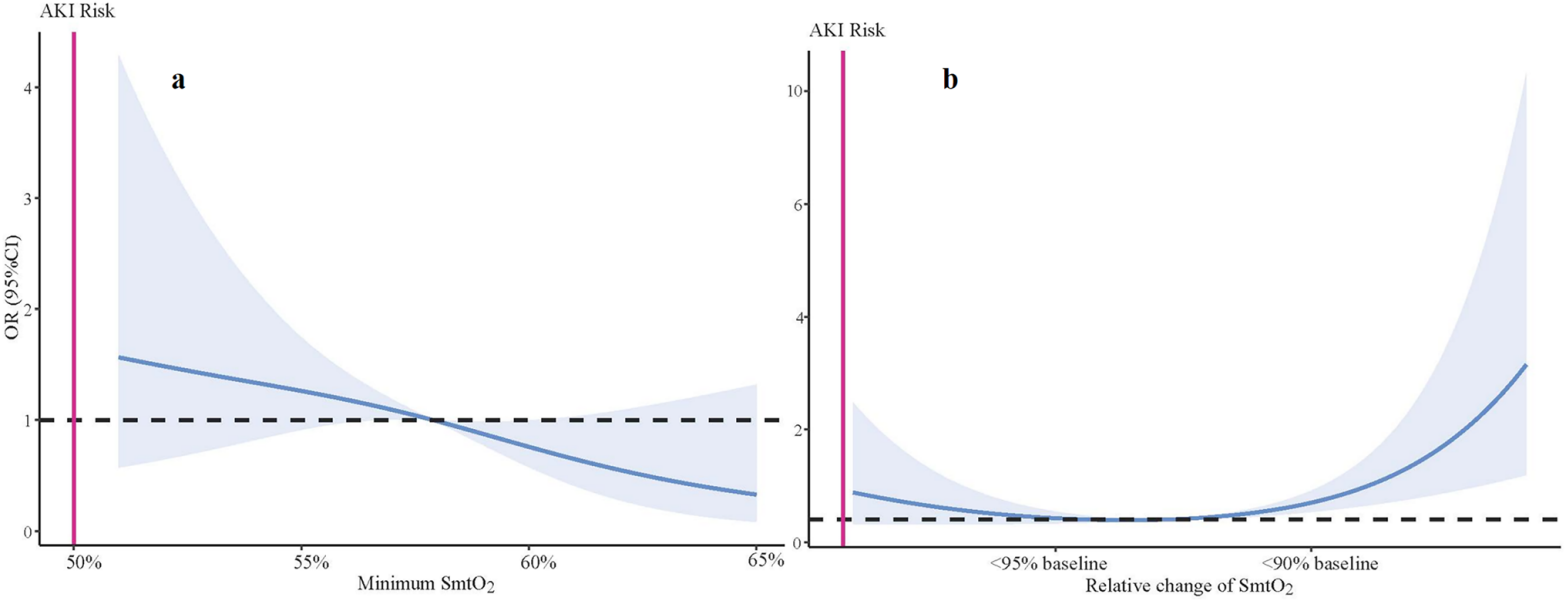
Unadjusted restricted cubic spline was utilized for identifying associations between SmtO_2_ at quadriceps and AKI. **a** Minimum SmtO_2_ at quadriceps was not associated with an increased risk of AKI with non-linear manner (*P*_overall_ = 0.072, *P*_*non*linear_ = 0.727). Minimum SmtO_2_ was defined as the absolute lowest value lasting for more than 60 s during surgery. **b** Relative change of SmtO_2_ at quadriceps was associated with an increased risk of AKI with non-linear manner (*P*_overall_ < 0.001, *P*_*non*linear_ = 0.002). Relative change of SmtO_2_ was calculated as the differences between baseline value and minimum SmtO_2_. The 95% CI of the odds ratio is represented by the shaded area. AKI, Acute kidney injury; SmO_2_, Muscular tissue oxygen saturation; OR, Odds ratio; CI, Confidence interval

RCS analysis showed that relative desaturation at quadriceps, but not minimum SmtO_2_, was associated with an increased risk of AKI with non-linear manner (*P*_overall_ < 0.001, *P*_*non*linear_ = 0.002), Fig. 2. By direct visualization of RCS curve, SmtO_2_ < 90% baseline at quadriceps was selected as threshold of desaturation. Additionally, the association between SmtO_2_ < 90% baseline and AKI was also tested in multivariable analysis (Supplemental file 1). The same method was also used to test the other different thresholds at quadriceps (i.e. < 95% baseline, > 105% baseline, and > 110% baseline) and AKI.

Patients were assigned into desaturation group or control group based on if they suffered any episode of muscular desaturation. The mean age of patients in desaturation group was 70.3 ± 4.6 which was comparable with 71.4 ± 5.1 in control group (*P* = 0.156). Baseline SmtO_2_ was 62.8 (60.9, 64.7) in desaturation group was also comparable with 62.9 (60.3, 65.1) in control group (*P* = 0.910), Table 1. Patients in desaturation group had longer duration of surgery time (*P* = 0.035), received more crystal fluid infusion (*P* = 0.011), lower MAP (*P* = 0.003), and higher SVV (*P* = 0.009). And these patients also had a higher incidence of intraoperative hypothermia (*P* = 0.028), Table 2.

**Table 1:** Patients demographic and preoperative variables with and without muscular desaturation

Variables^a^	Desaturation group^b^ (n = 68)	Control group (n = 168)	*P* value^c^
Age, yr	70.3 ± 4.6	71.4 ± 5.1	0.156
Male, n (%)	54 (79.4)	115 (68.5)	0.091
BMI, kg/m^2^	22.9 ± 3.6	23.6 ± 3.2	0.146
ASA, n (%)			0.808
II	30 (44.1)	77 (42.9)	
III	38 (55.9)	99 (56.5)	
IV	0 (0)	1 (0.6)	
Charlson Comorbidity Index	2 [2, 3]	2 [2, 3]	0.719
Mini nutritional assessment	11 [9, 13]	11 [10, 13]	0.273
Revised cardiac risk index	1 [1, 2]	1 [1, 2]	0.519
Comorbidity, n (%)			
Stroke	14 (20.6)	33 (19.6)	0.869
Hypertension	29 (42.6)	86 (51.2)	0.234
Coronary heart disease	18 (26.5)	52 (31.0)	0.495
Smoke	32 (47.1)	60 (35.7)	0.106
Diabetes	8 (11.8)	38 (22.6)	0.057
Chronic obstructive pulmonary diseases	8 (11.8)	25 (14.9)	0.532
Calcium channel blockers	16 (23.5)	41 (24.4)	0.887
Angiotensin receptor blockers	13 (19.1)	31 (18.5)	0.905
β-blocker	9 (13.2)	23 (13.7)	0.926
Statins	12 (17.6)	25 (14.9)	0.597
Hemoglobin, g/L	133 [113, 150]	129 [113, 146]	0.486
Albumin, g/L	37.4 [33.9, 39.6]	37.3 [33.9, 41.0]	0.484
Creatinine, μmol/L	64.0 [52.8, 78.5]	63.2 [54.3, 71.1]	0.667
Blood urea nitrogen, mmol/L	5.5 [4.8, 6.8]	5.0 [4.0, 6.1]	0.013
eGFR (ml/min/1.73m^2^)	98.7 [84.8, 108.2]	98.0 [82.8, 106.3]	0.479
Blood glucose, mmol/L	5.1 [4.7, 5.7]	5.3 [4.8, 6.2]	0.117
Arterial oxygen partial pressure, mmHg	69.7 [65.4, 76.5]	68.8 [64.4, 72.9]	0.197
SmtO_2_ baseline^d^, %			
Quadriceps	62.8 [60.9, 64.7]	62.9 [60.3, 65.1]	0.910
Left flank	65.5 [63.0, 67.1]	65.2 [63.4, 67.5]	0.473
Right flank	65.2 [62.8, 67.3]	65.4 [63.4, 67.7]	0.450

AKI, Acute kidney injury; ASA, American Society of Anesthesiologist; BMI, Body mass index; eGFR, Estimated glomerular filtration rate; SmtO_2_, Muscular tissue oxygen saturation

^a^Data are in mean ± SD or median [IQR] for continuous variables and in count (percentage) for binary variables

^b^Muscular desaturation was defined as SmtO_2_ at quadriceps < 90% baseline

^c^Unpaired t test or Kruskal–Wallis test was used for continuous variables and Chi-Squared test or Fisher exact test for binary variables

^d^SmtO_2_ baseline was measured with patients resting and breathing room air

**Table 2:** Patients intraoperative variables with and without muscular desaturation

Variables^a^	Desaturation group^b^ (n = 68)	Control group (n = 168)	*P* value^c^
Surgery type, n (%)			
Colorectal surgery	32 (47.1)	115 (68.5)	0.084
Gastric surgery	32 (47.1)	34 (20.2)	
Hepatic surgery	4 (5.9)	19 (11.3)	
Surgical time (min)	235 [175, 282]	209 [163, 252]	0.035
Propofol, mg	662 [535, 850]	650 [460, 835]	0.330
Sufentanil, μg	30 [25, 35]	30 [25, 35]	0.951
Rocuronium, mg	100 [88, 125]	100 [80, 120]	0.167
Remifentanil, mg	3.0 [2.3, 3.7]	2.8 [2.0, 3.4]	0.156
Etomidate, mg	16 [11, 20]	15 [10, 20]	0.354
Ephedrine, n (%)	20 (29.4)	54 (32.1)	0.682
Phenylephrine, n (%)	5 (7.4)	8 (4.8)	0.429
Norepinephrine, n (%)	15 (22.1)	32 (19.0)	0.600
Urapidil, n (%)	12 (17.6)	19 (11.3)	0.192
NSAIDs, n (%)	44 (64.7)	93 (55.4)	0.187
Crystal input, ml	2700 [2000, 2800]	2200 [1700, 2700]	0.011
Colloidal input, ml	250 [0, 500]	0 [0, 500]	0.320
Urine output, ml	400 [300, 600]	300 [200, 500]	0.121
Estimated blood loss, ml	100 [100, 200]	100 [100, 200]	0.250
Blood transfusion, n (%)	10 (14.7)	23 (13.7)	0.839
Hypotension^d^, n (%)	19 (27.9)	38 (22.6)	0.387
Minimum MAP, mmHg	68 [63, 72]	72 [67, 78]	0.003
Time-weighted MAP^e^, min	0 [0, 10]	0 [0, 0]	0.133
Maximum SVV	18 [16, 21]	16 [14, 19]	0.009
Hypothermia^f^, n (%)	21 (30.9)	30 (17.9)	0.028
Minimum hemoglobin, g/L	121 [101, 135]	119 [105, 133]	0.263
Maximum lactic acid, mmol/L	0.8 [0.7, 1.0]	0.8 [0.7, 1.1]	0.840
Minimum blood glucose, mmol/L	5.6 [4.9, 6.0]	5.4 [4.9, 6.3]	0.361
Minimum PO_2_, mmHg	136.0 [103.8, 188.5]	138.2 [109.2, 188.7]	0.583
Maximum PO_2_, mmHg	155.9 [122.3, 220.1]	174.7 [131.6, 224.0]	0.467

SmtO_2_, Muscular tissue oxygen saturation; MAP, Mean arterial pressure; SVV, Stroke volume variation; NSAIDs, Nonsteroidal anti-inflammatory drugs; PO_2_, Arterial oxygen partial pressure

^a^Data are in mean ± SD or median [IQR] for continuous variables and in count(percentage) for binary variables

^b^Muscular desaturation was defined as SmtO_2_ at quadriceps < 90% baseline

^c^Unpaired t test or Kruskal–Wallis test was used for continuous variables and Chi-Squared test or Fisher exact test for binary variables

^d^Hypotension was defined as MAP < 60 mmHg that required treatments throughout surgery

^e^Time-weighted MAP was cumulative time with MAP below 60 mmHg

^f^Hypothermia was defined as nasopharyngeal temperature < 36℃ during surgery

### Primary outcome

The overall incidence of AKI was 18.6% (44/236), with 70.5% (31/44) of cases occurring within the first 48 h postoperatively. Among AKI cases, stage 1 and stage 2 AKI accounted for 84.1% (37/44) and 15.9% (7/44), respectively. No patients experienced stage 3 AKI or required dialysis.

### Association between SmtO_2_ at quadriceps and AKI

The incidence of relative desaturation (SmtO_2_ at quadricep < 90% baseline) was 28.8% (68/236). In desaturation group, the occurrence of AKI was 27.9% (19/68), significantly higher than 14.9% (25/168) in control group (RR = 2.22, 95% CI 1.26–4.37, *P* = 0.020). No statistically significant differences were observed in stage 1 (*P* = 0.163) or stage 2 (*P* = 0.088) of AKI between the two groups.

Seven factors were potentially associated with AKI including age (*P* = 0.022), ASA classification (*P* = 0.002), coronary heart disease (*P* = 0.007), surgical duration (*P* = 0.047), intraoperative maximum SVV (*P* = 0.034), postoperative use of diuretics (*P* = 0.037), and ICU admission (*P* = 0.001), Supplemental file 2. After adjustment of above confounders, relative desaturation at quadricep was associated with an increased risk of AKI (OR = 2.84, 95% CI 1.21–6.67, *P* = 0.016).

The minimum SmtO_2_ at quadricep was 56.8 (54.2–60.1) in desaturation group and 58.2 (54.7–61.0) in control group, (*P* = 0.219). After multivariable analysis, it was not found to be associated with AKI (OR = 0.99, 95% CI 0.91–1.09, *P* = 0.906), Table 3.

**Table 3:** Association between muscular desaturation and AKI

Variables^a^	Multivariate logistic regression^c^	
	OR (95% CI)	*P* value
Muscular desaturation^b^ (yes versus no)	2.84 (1.21–6.67)	0.016
Age (per year increase)	1.02 (0.94–1.11)	0.617
ASA (per level increase)	3.28 (1.16–9.31)	0.025
Coronary heart disease (yes versus no)	2.08 (0.81–5.32)	0.158
Surgical duration (per min increase)	1.01 (0.99–1.01)	0.302
Maximum SVV (per one increase)	1.11 (1.01–1.22)	0.025
Postoperative use of diuretics (yes versus no)	2.27 (0.79–6.55)	0.130
ICU (yes versus no)	1.99 (0.84–4.72)	0.120

AKI, Acute kidney injury; SmtO_2_, Muscular tissue oxygen saturation; ASA, American Society of Anesthesiologist; SVV, Stroke volume variation; ICU, Intensive care unit

^a^The confounders included in this multivariable logistic regression are age, ASA, coronary heart disease, surgical time, maximum SVV, postoperative use of diuretics and ICU admission

^b^Muscular desaturation was defined as SmtO_2_ at quadriceps < 90% baseline

^c^When using the minimum SmtO_2_ measured at quadriceps in multivariable logistic regression, the OR is 0.99 (95% CI 0.91–1.09; *P* = 0.906)

### Association between SmtO_2_ at flanks and AKI

The incidences of relative desaturation (i.e., < 90% baseline) at left and right flanks were 6.8% and 3.8%, respectively. In multivariable analysis, relative desaturation at left flank (OR = 6.38, 95% CI 1.78–22.89, *P* = 0.004) and right flank (OR = 8.90, 95% CI 1.42–45.63; *P* = 0.019) were both associated with an increased risk of AKI. The minimum SmtO_2_ at both sides of flanks were not associated with AKI (OR = 1.01, 95% CI 0.92–1.09; *P* = 0.938 and OR = 1.02, 95% CI, 0.91–1.14; *P* = 0.742, respectively), Supplemental file 3.

### Association between the AUC of SmtO_2_ and AKI

The AUC of SmtO_2_ at the quadriceps, < 90% baseline, showed an association with AKI (OR = 1.02; 95% CI 1.01–1.04; *P* = 0.014). Similarly, the AUCs of left and right flank SmtO_2_, < 90% baseline, were also associated with AKI (OR = 1.02; 95% CI 1.01–1.02;* P* = 0.008 and OR = 1.09; 95% CI 1.01–1.17; *P* = 0.025). None of the other AUCs calculated for relative changes based on the thresholds showed an association with AKI (Supplementary file 4).

### Other complications

In comparison to control group, patients in desaturation group exhibited a higher incidence of pulmonary infection (*P* = 0.048), atelectasis (*P* = 0.027), sepsis (*P* = 0.038), stroke (*P* = 0.026), and lower quality of recovery scores (*P* = 0.019) at 30-day follow-up, Table 4.

**Table 4:** Other postoperative complications with and without muscular desaturation

Complications	Desaturation group (n = 68)	Control group (n = 168)	*P* value^a^
Pulmonary infection	22 (32.4)	34 (20.2)	0.048
Respiration failure	10 (14.7)	12 (7.1)	0.070
Atelectasis	12 (17.6)	13 (7.8)	0.027
Arrhythmia	5 (7.4)	4 (2.4)	0.071
Heart failure	1 (1.5)	0 (0)	0.115
Myocardial infarction	0 (0)	1 (0.6)	0.524
Stroke	2 (2.9)	0 (0)	0.026
Pulmonary embolism	9 (13.4)	13 (7.7)	0.176
Deep vein thrombosis	6 (8.8)	9 (5.4)	0.323
Acute hepatic injury	2 (2.9)	10 (6.0)	0.340
Incision infection	2 (2.9)	18 (10.7)	0.052
Sepsis	4 (5.9)	2 (1.2)	0.038
Postoperative hospitalization duration	11 [8, 15]	10 [8, 13]	0.103
30d-Rehospitalization	4 (5.9)	15 (9.0)	0.423
30d-Mortality	4 (5.9)	5 (3.0)	0.291
15-item quality of recovery scale	89 [73, 100]	97 [85, 103]	0.019

^a^Data are in median [IQR] for continuous variables and in count (percentage) for binary variables

Kruskal–Wallis test was used for continuous variables and Fisher exact test for binary variables

## Discussion

Our study re-confirmed that AKI was a prevalent complication among elderly patients following major non-cardiac surgery. Notably, we identified that muscular desaturation, SmtO_2_ < 90% baseline at quadriceps or flanks, was significantly associated with an increased risk of AKI.

In present study, the incidence of AKI was about 18% which was slightly higher than the results of previous studies [1, 8, 22]. This higher incidence can be attributed to several factors. First, our study focused on elderly patients, with a mean age of 71 ± 5, a known risk factor for AKI [23]. Secondly, our cohort exhibited a higher prevalence of risk factors for AKI, including ASA Class III (56.4%), coronary heart disease (29.7%), ICU admission (23.7%), and prolonged surgical durations (244 ± 100 min) [24–26]. In fact, more than one-third of our patients had four or more risk factors, as assessed by the AKI index, a widely used tool for AKI risk assessment [27]. Thirdly, our diligent monitoring of serum creatinine levels over a 7-day period enabled us to detect late-onset AKI occurrences between 48 h and 7 days postoperatively. This is of paramount importance, as late-onset AKI has been associated with a higher 3-year mortality rate when compared to early-onset AKI [28].

The preservation of tissue oxygenation holds pivotal significance for maintaining organ function homeostasis [18]. The kidney, in particular, is susceptible to hemodynamic disturbances during anesthesia and surgery due to its disrupted pressure autoregulation [29]. The challenge lies in precisely, promptly, and non-invasively monitoring the balance between oxygen supply and consumption in the kidney. Although near-infrared spectroscopy (NIRS) has been validated for monitoring renal oxygenation in pediatric patients undergoing cardiac surgery and critically ill infants, [30, 31] its application in adults is constrained by the deeper location of the kidney beneath layers of tissue (6–8 cm deep) [32]. To address this limitation, some studies used ultrasound to screen adult patients with kidneys located less than 4 cm from the skin. [21, 33, 34] However, this method does not fully account for potential interference from other tissue oxygenation (e.g., muscular oxygenation) on the accuracy of kidney monitoring.

Considering the formidable task of directly detecting kidney oxygenation, our present study examined the affirmative association between muscular desaturation at the quadriceps and AKI. This underscores the potential utility of muscular oxygenation as an indicator for predicting AKI. Previous studies have indicated that SmtO_2_ values below specific thresholds, such as SmtO_2_ < 66% at the forearm in a liver transplantation cohort or SmtO_2_ < 54.5% at the thenar muscle in cardiac surgery patients, are associated with the development of AKI [35–37]. Our study further contributes to the evidence supporting the use of SmtO_2_ in predicting AKI in elderly patients following non-cardiac surgery. However, this present study was exploratory. We continuously monitored bilateral franks and quadriceps SmtO_2_ for timely predicting postoperative AKI based on previous studies [18, 19, 36] and for enhanced convenience of intraoperative monitoring. Additional researches are required at alternative monitoring sites as well as underlying pathological and physiological mechanisms associated with reduction in SmtO_2_ and postoperative AKI.

However, it is imperative to acknowledge that SmtO_2_ can be calculated using various parameters, and its values may vary significantly among different populations and outcomes. Previous investigations have reported associations between SmtO_2_ and clinical outcomes by employing various SmtO_2_ parameters, encompassing the minimum value, absolute value, and area under the curve (AUC) below a specific threshold. [38, 39] For instance, SmtO_2_ < 75% has been related to organ failure in sepsis patients, while the minimum SmtO_2_ at the thenar eminence was inversely associated with poor outcomes after major non-cardiac surgery [40, 41]. The AUC of SmtO_2_ has also demonstrated a significant correlation with the length of hospital stay following major spine surgery [18]. In our study, we discovered that the minimum SmtO_2_ at any tissue site did not associate with AKI. Unlike the minimum SmtO_2_, the relative change in SmtO_2_ may more accurately reflect individual shifts in muscular saturation. For instance, 19.1% of our enrolled patients exhibited a baseline SmtO_2_ below 55%, potentially leading to misclassification as "desaturation" when applying an absolute threshold of 55%. Our study showed that AUCs of quadriceps, biliteral franks for reductions of 10% from the baseline were associated with increasing odds of AKI, which suggested the duration and degree of SmtO_2_ change over the threshold may related to AKI. But calculating the AUC, is practically challenging and cannot be computed promptly in a prospective manner [38].

Our study also revealed that patients who experienced muscular desaturation may result in a higher incidence of other postoperative complications. Tissue hypoxia frequently manifests during surgical procedures and may result in organ dysfunction and unfavourable outcomes [39]. Previous investigations have associated SmtO_2_ values below specific thresholds with various postoperative complications, including pulmonary infection, postoperative nausea and vomiting (PONV), and ICU mortality [19, 42, 43]. Nevertheless, the efficacy of interventions guided by SmtO_2_ in preventing these complications remains a topic of debate. Some studies have reported mixed results concerning the effectiveness of maintaining specific SmtO_2_ thresholds in improving clinical outcomes. In a multicentre randomized controlled study, maintaining intra-operative SmtO_2_ at or above baseline or 70% at flank muscle did not correlate with a reduced risk of PONV in patients undergoing hysterectomy [44]. In another study, maintaining SmtO_2_ > 80% in the forearm did not enhance clinical outcomes but prolonged mechanical ventilation and increased the likelihood of red blood cell transfusions [45]. A pilot study similarly reported that setting SmtO_2_ ≥ 80% as a target did not reduce the incidence of postoperative complications or the length of ICU stay following high-risk surgery [46]. Consequently, further investigations are warranted to identify specific SmtO_2_ thresholds and assess their impact on distinct outcomes and patient populations.

Our study had several limitations. First, the diagnosis of AKI was based solely on serum creatine without urine output. Although this method was adopted by most studies, it might underestimate the incidence of AKI [13, 30]. Second, the underlying mechanisms linking muscular desaturation and AKI remain unclear. During episodes of hypoperfusion, the body prioritizes perfusion to vital organs such as the brain and kidneys, potentially sacrificing peripheral tissues like skin and muscle during hemodynamic instability [29]. Thus, muscular desaturation may serve as an early indicator of hypo-perfusion. Third, further studies are required to verify whether interventions guided by SmtO_2_ can effectively mitigate the observed complications.

## Conclusion

Muscular desaturation was associated with an increased risk of AKI in elderly patients after major abdominal surgery. This finding suggests that SmtO_2_ may serve as a potential indicator for predicting AKI.

### Supplementary Information

Below is the link to the electronic supplementary material.

Supplementary file1 (DOCX 13 KB)

Supplementary file2 (DOCX 15 KB)

Supplementary file3 (DOCX 16 KB)

Supplementary file4 (DOCX 14 KB)

## Data Availability

The data that support the findings of this study are available on request from the corresponding author, [Xinli Ni], upon reasonable request.
