# Supplementary material for: Association between muscular tissue desaturation and acute kidney injury in older patients undergoing major abdominal surgery: a prospective cohort study
Source: J Anesth. 2024 Apr 6;38(4):434–44. doi: 10.1007/s00540-024-03332-6 (PMC11284187; doi:10.1007/s00540-024-03332-6)
Supplement: Supplementary file 1 — Supplementary file1 (DOCX 13 KB) [file 540_2024_3332_MOESM1_ESM.docx]

| **Supplementary file 1. Incidence of relative changes of SmtO_2_ at quadriceps and their association with AKI** | | | | | | |
| --- | --- | --- | --- | --- | --- | --- |
| **Threshold** | **Exceeding Threshold** | **Not Exceeding Threshold** | **Univariate logistic regression** | | **Multivariable logistic regression ^b^** | |
|  | **AKI/Total patient No. (%) ^a^** | | **OR (95% CI)** | ***P* value** | **OR (95% CI)** | ***P* value** |
| < 90 % baseline | 19/68 (27.9) | 25/168 (14.9) | 2.89 (1.45 - 5.76) | 0.021 | 2.84 (1.21 - 6.67) | 0.016 |
| < 95% baseline | 29/151 (19.2) | 15/85 (17.6) | 1.11 (0.56 - 2.21) | 0.768 | 0.84 (0.37 - 1.92) | 0.678 |
| > 105% baseline | 26/130 (20.0) | 18/106 (17.0) | 1.22 (0.63 - 2.38) | 0.554 | 1.43 (0.65 - 3.16) | 0.374 |
| > 110% baseline | 8/42 (19.0) | 36/194 (18.6) | 1.03 (0.44 - 2.42) | 0.941 | 0.95 (0.32 - 2.76) | 0.919 |

Abbreviations: AKI, Acute kidney injury. SmtO_2_, muscular tissue oxygen saturation.

a, The numerator is the number of patients with AKI while the denominator is the number of patients who below or not below the threshold.

b, Each threshold was adjusted with confounders in including age, ASA, coronary heart disease, surgical duration, maximum SVV, postoperative use of diuretics and ICU admission.
