# Supplementary material for: Association between muscular tissue desaturation and acute kidney injury in older patients undergoing major abdominal surgery: a prospective cohort study
Source: J Anesth. 2024 Apr 6;38(4):434–44. doi: 10.1007/s00540-024-03332-6 (PMC11284187; doi:10.1007/s00540-024-03332-6)
Supplement: Supplementary file 2 — Supplementary file2 (DOCX 15 KB) [file 540_2024_3332_MOESM2_ESM.docx]

| **Supplementary file 2. Risk factors of AKI identified by Univariate logistic regression** | | |  |
| --- | --- | --- | --- |
| **Variables** | **OR (95% CI)** | ***P* value** | |
| Age | 1.08 (1.01-1.15) | 0.022 | |
| Male | 0.59 (0.27-1.32) | 0.199 | |
| BMI | 1.08 (0.97-1.19) | 0.149 | |
| ASA | 3.33 (1.57-7.04) | 0.002 | |
| Charlson Comorbidity Index | 1.10 (0.94-1.28) | 0.239 | |
| Mini nutritional assessment | 0.89 (0.84-1.33) | 0.169 | |
| Revised cardiac risk index | 1.40 (0.92-2.13) | 0.121 | |
| Hypertension | 1.33 (0.69-2.57) | 0.393 | |
| Coronary heart disease | 2.53 (1.29-4.95) | 0.007 | |
| Stroke | 1.04 (0.46-2.35) | 0.921 | |
| Chronic obstructive pulmonary diseases | 1.48 (0.62-3.56) | 0.376 | |
| Pre-Hemoglobin | 1.01 (0.99-1.02) | 0.482 | |
| Pre-Albumin | 0.98 (0.92-1.04) | 0.488 | |
| Pre-Blood urea nitrogen | 0.92 (0.76-1.12) | 0.409 | |
| Surgical duration | 1.02 (1.01-1.02) | 0.047 | |
| Hypothermia **^a^** | 1.08 (0.49-2.38) | 0.842 | |
| Hypotension **^b^** | 1.06 (0.50-2.26) | 0.884 | |
| Minimum MAP | 0.98 (0.94-1.03) | 0.443 | |
| Maximum SVV | 1.09 (1.01-1.18) | 0.034 | |
| Crystal input | 1.00 (0.98-1.01) | 0.113 | |
| Blood transfusion | 0.97 (0.37-2.50) | 0.941 | |
| Postoperative minimum Hb | 0.98 (0.97-1.19) | 0.161 | |
| Postoperative minimum Alb | 0.93 (0.86-1.02) | 0.114 | |
| Postoperative contrast agent | 1.53 (0.68-3.41) | 0.301 | |
| NASIDs | 1.18 (0.61-2.32) | 0.622 | |
| Postoperative use of diuretics | 2.39 (1.05-5.43) | 0.037 | |
| ICU | 3.18 (1.59-6.39) | 0.001 | |

Abbreviations: AKI, Acute kidney injury; ASA, American Society of Anesthesiologist; BMI, Body mass index; MAP, mean arterial pressure; SVV, Stroke volume variation; NSAIDs, Nonsteroidal anti-inflammatory drugs; ICU, Intensive care unit.

a, Hypothermia was defined as nasopharyngeal temperature < 36℃ during surgery.

b, Hypotension was defined as MAP < 60 mmHg that required treatments throughout surgery.
