# Supplementary material for: Association between muscular tissue desaturation and acute kidney injury in older patients undergoing major abdominal surgery: a prospective cohort study
Source: J Anesth. 2024 Apr 6;38(4):434–44. doi: 10.1007/s00540-024-03332-6 (PMC11284187; doi:10.1007/s00540-024-03332-6)
Supplement: Supplementary file 3 — Supplementary file3 (DOCX 16 KB) [file 540_2024_3332_MOESM3_ESM.docx]

| **Supplementary file 3. Incidence of relative changes of SmtO_2_ at flanks and their association with AKI** | | | | | | |
| --- | --- | --- | --- | --- | --- | --- |
| **Thresholds ^a^** | **Exceeding Threshold** | **Not Exceeding Threshold** | **Univariate logistic regression** | | **Multivariable logistic regression ^c^** | |
|  | **AKI/Total patient No. (%) ^b^** | | **OR (95% CI)** | ***P* value** | **OR (95% CI)** | ***P* value** |
| **Left flank SmtO_2_** | | | | | | |
| < 90% baseline | 9/16 (56.3) | 35/220 (16.2) | 6.65 (2.32 - 19.04) | <0.001 | 6.38 (1.78 - 22.89) | 0.004 |
| < 95% baseline | 11/45 (24.4) | 33/187 (17.6) | 1.51 (0.69 - 3.28) | 0.299 | 1.10 (0.44 - 2.71) | 0.401 |
| > 105% baseline | 31/140 (22.1) | 13/96 (19.7) | 1.27 (0.78 - 2.76) | 0.136 | 2.19 (0.92 - 5.19) | 0.076 |
| > 110% baseline | 9/32 (28.1) | 35/200 (17.5) | 1.85 (0.79 - 4.33) | 0.159 | 1.79 (0.67 - 4.79) | 0.245 |
| **Right flank SmtO_2_** |  |  |  |  |  |  |
| < 90% baseline | 6/9 (66.7) | 37/224 (16.5) | 10.11 (2.42 - 42.24) | 0.002 | 8.90 (1.42 - 45.63) | 0.019 |
| < 95% baseline | 8/26 (30.8) | 35/207 (16.9) | 2.20 (0.88 - 5.42) | 0.092 | 1.52 (0.52 - 4.44) | 0.446 |
| > 105% baseline | 28/139 (20.1) | 15/94 (16.0) | 1.33 (0.67 - 2.65) | 0.420 | 1.83 (0.79 - 4.23) | 0.157 |
| > 110% baseline | 6/24 (25.0) | 37/209 (17.7) | 1.55 (0.58 - 4.17) | 0.386 | 1.79 (0.56 - 5.76) | 0.329 |

Abbreviations: AKI, Acute kidney injury. SmtO_2_, muscular tissue oxygen saturation.

a, Each threshold was adjusted with confounders in including age, ASA, coronary heart disease, surgical duration, maximum SVV, postoperative use of diuretics and ICU admission.

b, The numerator is the number of patients with AKI while the denominator is the number of patients who below or not below the threshold.

c, When using the minimum SmtO_2_ measured at left and right flank muscles in multivariable logistic regression, the OR is 1.01 (95% CI, 0.92-1.09; *P* = 0.938) and 1.02 (95% CI, 0.91-1.14; *P* = 0.742), respectively.
