# Supplementary material for: Association between muscular tissue desaturation and acute kidney injury in older patients undergoing major abdominal surgery: a prospective cohort study
Source: J Anesth. 2024 Apr 6;38(4):434–44. doi: 10.1007/s00540-024-03332-6 (PMC11284187; doi:10.1007/s00540-024-03332-6)
Supplement: Supplementary file 4 — Supplementary file4 (DOCX 14 KB) [file 540_2024_3332_MOESM4_ESM.docx]

| **Supplementary file 4. Association between each AUC of set threshold and AKI** | | |
| --- | --- | --- |
| **Threshold used for AUC calculation ^a^** | **Multivariable logistic regression ^b^** | |
|  | **OR (95% CI) Every AUC increase** | ***P* value** |
| **Quadriceps SmtO_2_** |  |  |
| < 90% baseline | 1.023 (1.005,1.042) | 0.014 |
| < 95% baseline | 1.006 (1.002,1.010) | 0.004 |
| > 105% baseline | 1.001 (0.998,1.003) | 0.687 |
| > 110% baseline | 1.001 (0.993,1.010) | 0.772 |
| **Left flank SmtO_2_** |  |  |
| < 90% baseline | 1.005(0.989,1.022) | 0.527 |
| < 95% baseline | 1.001 (0.996,1.007) | 0.678 |
| > 105% baseline | 1.002 (1.000,1.004) | 0.048 |
| > 110% baseline | 1.001 (0.997,1.005) | 0.577 |
| **Right flank SmtO_2_** |  |  |
| < 90% baseline | 1.088 (1.011,1.171) | 0.025 |
| < 95% baseline | 1.017 (1.001,1.033) | 0.041 |
| > 105% baseline | 1.002 (0.999,1.004) | 0.147 |
| > 110% baseline | 1.004 (0.996,1.012) | 0.287 |

Abbreviations: AKI, Acute kidney injury; SmtO_2_, muscle tissue oxygen saturation. AUC, area under curve
a, AUCs (min × %) were calculated as the size of the area which exceeded the threshold defined by the relative change.
b, Each threshold was adjusted with confounders in including age, ASA, coronary heart disease, surgical duration, maximum SVV, postoperative use of diuretics and ICU admission.
